# Identification of ectoparasitic insects among domestic goats in Bulgaria

**DOI:** 10.14202/vetworld.2023.728-734

**Published:** 2023-04-12

**Authors:** Nikola Stefanov Nizamov

**Affiliations:** Department of Veterinary Microbiology, Infectious and Parasitic Diseases, Faculty of Veterinary Medicine, Trakia University, Stara Zagora 6014, Bulgaria

**Keywords:** Bulgaria, ectoparasites, fleas, goats, lice, prevalence

## Abstract

**Background and Aim:**

Ectoparasitic entomoses cause serious economic losses to small-scale farmers. Parasites have both direct and indirect impacts on hosts. Domestic goats are a common target of infestation with ectoparasitic insects. This study aimed to identify the species of ectoparasitic insects in domestic goats in Bulgaria.

**Materials and Methods:**

The study was performed in 34 farms from 29 settlements in 16 regions of Bulgaria. A total of 4599 goats from eight breeds, naturally infested with ectoparasitic insects were included in the study. The goats were inspected with a magnifying glass for the presence of skin changes (alopecia, dandruff, crusts, and nodules), eggs and adult ectoparasites. The detected insects were collected individually with tweezers and preserved in containers with 70% ethanol. Over the study period, 5,651 insects were collected; their species, sex, and developmental stage were identified by their morphological features and biometric measurements.

**Results:**

Six species from 5 genera were detected: *Linognathus stenopsis* Burmeister, 1838; *Linognathus africanus* Kellog and Paine, 1911; *Bovicola caprae* Gurlt, 1843; *Pulex irritans* Linnaeus, 1758; *Hippobosca equina* Linnaeus, 1758; and *Lipoptena cervi* Linnaeus, 1758. *Linognathus stenopsis* were the most prevalent, followed by *B. caprae* and *P. irritans*. In detected lice populations, female insects were more numerous; female: male ratios ranged from 2.2 to 7.2 and imagines prevailed over the nymphs. In fleas, male imagines were more numerous than females (1:0.8).

**Conclusion:**

The study demonstrated that the species *L. stenopsis*, *L. africanus*, *B. caprae*, and *P. irritans* were encountered in more than 40% of surveyed farms, situated in 68.75% of regions in Bulgaria. The most intense infestation was by species from the *Linognathus* genus (907 insects), whereas the highest extensity of infestation was registered for *P. irritans* (32.3%). This study detected *P. irritans* as the only flea species.

## Introduction

Ectoparasitic entomoses cause serious economic losses to small-scale farmers. Parasites have both direct and indirect impacts on hosts. The direct effects are due to blood loss, skin inflammation and irritation, as well as toxic effects and allergic response, whereas the indirect impact comprises anxiety, disturbed feeding and rest, self-mutilation of pruritic animals [1]. Domestic goats are a common target of infestation with ectoparasitic insects from orders Phthiraptera, Siphonaptera, and Diptera.

Phthirapterosis in goats is mainly caused by species from the genera *Linognathus* and *Bovicola* (*Damalinia*) [2]. The *Linognathus* species *Linognathus stenopsis* Burmeister, 1838 and *Linognathus africanus* Kellog and Paine, 1911 are specific for domestic goats. Three *Bovicola* species are also species-specific: *Bovicola caprae* in goats, *Ballota limbata*, and *B. crassipest* in Angora goats. Touleshkov [3] and Nedelchev [4] reported two lice species infesting domestic goats in Bulgaria - *L. stenopsis* and *B. caprae*. The following fleas from order Siphonaptera were reported to infest goats: *Ctenocephalides canis* Curtis, 1826 [5], *Pulex irritans* Linnaeus, 1758 [6, 7], and *Ctenocephalides felis* Bouche, 1835 [8]. According to Soulsby [9] and Bezerra-Santos [10], species from the family Hippoboscidae infesting domestic goats were *Hippobosca equina* Linnaeus, 1758, *Lipoptena cervi* Linnaeus, 1758 and *Melophagus ovinus* Linnaeus, 1857.

Data about the population structure of lice-infesting goats are scarce. According to Santos *et al*. [11], the sex-related prevalence of *B. caprae* and *L. africanus* was similar with respect to male to female insect ratio (*B. caprae*: 1:4.8; *Linognathus africanus*: 1:4.6). The analysis of age structure of fleas is difficult due to the free living stages (larva, pupa, and preimago), and deposition of eggs not only on the host but in the environment as well [12].

The extensity of infestation (EI) measured in percentage gives information on the spread of the parasite amongst the examined population of animals, indicating the proportion of infested animals of all investigated subjects. The high EI of Phthiraptera and Siphonaptera infestation suggests that they are a serious concern for goat farms [13, 14]. In our country, Nedelchev [4] reported that out of 35 surveyed farms, 32 were infested with lice: 7 – only with *B. caprae*, 3 – only with *L. stenopsis*, and mixed infestation with both species was detected in 22 farms.

The intensity of infestation (II) is the number of parasitic individuals of a certain species (determined directly or indirectly) found on a sole host [15]. In highly mobile species as fleas, where the exact II cannot be determined, the degree of infestation (DI) is used instead of II. The degree of infestation gives information on the quantity of the parasites: severe, medium, weak, or absent. These characteristics of the infestation process are of critical importance as they determine the presence of illness (in cases of high parasite counts) or just hosting (in cases of low parasite counts). Data about the II of lice parasitizing on goats are rather scarce. Horak *et al*. [16] found a mean II with *Damalinia limbata* of 295 in one-week-old goat kids that increased to 3,392 a month later. The same authors reported II with *L. africanus* exceeding 12,000 insects in one-month-old goat kids. According to Fomicheva [17], the mean II in goats infested with *B. caprae* ranged from 189 to 296. The extreme motility of fleas makes the determination of II very difficult, which is probably the cause of the lack of literature data. According to Christodoulopoulos and Theodoropoulos [6] and Christodoulopoulos *et al*. [7], DI with *P. irritans* in domestic goats and goat kids in Greece was 7–10 insects in adult goats and 10–15 insects in goat kids.

So far, such a large-scale study in our country has not been conducted. The obtained results will throw light on the seriousness of the problem with ectoparasitic insects in goats and help to build prevention strategies. That would lead to containment of their spread to minimize losses due to reduced productivity and death of the animals.

This study aimed to determine the species composition and spread of ectoparasitic insects in domestic goats in Bulgaria and to present detailed information on their population structure, intensity and EI.

## Materials and Methods

### Ethical approval and Informed consent

Ethical approval was not required for this study; however, each animal was examined without any harm to the animals. Verbal consent was obtained from the owners before the study.

### Study period and location

The study was conducted from January 2018 to December 2019 in 34 farms from 29 settlements in 16 regions of Bulgaria. It included 4,599 domestic goats belonging to eight breeds naturally infested with ectoparasitic insects. The farms were located in regions with various altitude. The prevailing climate in Bulgaria is transitional, between temperate continental and continental Mediterranean. The average annual temperature in the country is between 10°C and 14°C. The average January temperature is −1°C and the average July temperature: 24°C–26°C. The average annual precipitation is 550–600 mm.

### Herds studied

The visit to the farms was paid after the owners alarmed for strong anxiety and pruritus in goats. Before the examination, the owner (caretaker) of animals was requested to fill in a questionnaire giving information about the housing technology, veterinary services, parasitological problems, and anti-parasitic treatment of the herd. In all herds, the animals were reared in a mixed farming system: From spring until autumn, the goats grazed on pasture and during the winter, they were housed indoor.

### Examination methods

One-tenth (10%) of the animals in each herd were examined as per the methods described in previous studies [18, 19]. The goats were inspected with a magnifying glass for the presence of skin changes (alopecia, dandruff, crusts, and nodules), eggs, and adult ectoparasites. The detected insects were collected individually with tweezers and were preserved in containers with 70% ethanol. All detected insects were transported to the laboratory in a plastic container at 4°C. A DMi1 S/M 424790 Leica® microscope (Leica Microsystems CMS GmbH, Wetzlar Germany) was used for the microscopic examinations (magnification 40X).

The identification of ectoparasitic species was done according to Price and Graham [20]; Wall and Shearer [21]; Hutson [22].

The shape of the head in *L. stenopsis* was elongated, whereas in *L. africanus* it had a prominent ocular bulging. The female gonopods of *L. africanus* were round and lacked the process described in *L. stenopsis*. In male *L. africanus* the gonopods had a pair of terminal tubercules with setae. The male gonopods of *B. caprae* were cone-shaped, densely covered with short bristles. The species had two terminal flaps close to the genital opening [20].

The head of *P. irritans* was round without ctenidia, but had a distinct ocular seta. The genal margin was supplied with a much reduced spine [21].

The species identification of *H. equina* was based on a pale median patch on the scutellum extending from the prescutum to the mesoscutum as well as the presence of a pale border on the prescutum and apico-lateral corners of the mesoscutum. Pale medium bands were present on the tibia of the mid and hind pair or legs [22].

The II with lice was determined using the method of Brown *et al*. [23] by counting all parasites in a total number of 7 square-shaped areas of 10 cm^2^ and multiplying the sum of all parasites by 100. The type of infestation was determined in the laboratory according to number, species, sex and developmental stage of insects.

The DI with fleas was established by the system of Christodoulopoulos *et al*. [7]. The animal was placed on its back and the fleas were counted on the least haired parts – abdomen, udder, perineal area, and medial side of the thighs. The interpretation was as follows: Level 0: no fleas (absence of infestation); Level 1: 1–2 fleas (very weak infestation); Level 2: 3–6 fleas (weak infestation); Level 3: 7–10 fleas (moderate infestation); Level 4: 11–15 fleas (severe infestation); and Level 5: 16 and more fleas (very severe infestation).

The EI for each parasitic species was determined by examination of goats and detection of the number of infested animals in each herd.

### Statistical analysis

The 95% confidence intervals for numbers and relative proportions of ectoparasitic insect species and for regional distribution of insect species in Bulgaria were calculated by routine methods of descriptive statistics with IBM^®^ SPSS^®^ statistics 26.0 software (IBM Corp., NY, USA) [24].

## Results

A total of 5651 insects were collected from naturally infested goats and their species, sex, and developmental stage were determined. Six species from three orders were detected: Order Phthiraptera with three species (*L. stenopsis* Burmeister, 1838, *L. africanus* Kellog and Paine, 1911, and *B. caprae* Gurlt, 1843); Order Siphonaptera with a single species (*P. irritans* Linnaeus, 1758) and Order Diptera with two species (*H. equina* Linnaeus, 1758 and *L. cervi* Linnaeus, 1758).

The analysis of data showed that out of the six species infesting domestic goats in Bulgaria, *L. stenopsis* was the most prevalent, followed by *B. caprae* and *P. irritans* (Table-1).

**Table-1:** Relative proportion of parasitic insect species infesting domestic goats in Bulgaria n = 5651.

Species	Detected number	Percentage	Confidence interval
*L. stenopsis*	3082	54. 53	53 ÷ 56
*L. africanus*	493	8. 72	7 ÷ 9
*B. caprae*	1412	24. 98	24 ÷ 26
*P. irritans*	566	10. 01	9 ÷ 11
*H. equina*	64	1. 13	0.5 ÷ 1.5
*L. cervi*	34	0. 60	0.4 ÷ 0.8

The data about *L. africanus* and *L*. *cervi* were presented in previous studies of ours [25, 26]; therefore, only data for the other four ectoparasitic species will be commented.

The population structure of lice and flea species infesting domestic goats in Bulgaria is presented in Table-2. Female insects were more numerous than males for all lice species with ratios ranging from 2.2 to 7.2. The opposite relationship was noted for fleas, where males prevailed over females (1:0.8).

**Table-2:** Age- and sex-related distribution of lice and flea species infesting domestic goats in Bulgaria.

Species	M: F	A: N	M: N	F: N
*L. stenopsis*	1: 3.5	1: 0.45	1: 2.0	1: 0.58
*L. africanus*	1: 2.2	1: 0.46	1: 1.5	1: 0.67
*B. caprae*	1: 7.2	1: 0.29	1: 2.4	1: 0.33
*P. irritans*	1: 0.8	-	-	-

M=Male; F=Female; A=Adults; N=Nymphs.

The number of imagines (imaginal stages) exceeded that of nymphal stages, but sex distribution demonstrated that the number of females was greater than that of nymphs, whereas the number of males: Lower than nymphs’ number. *Bovicola caprae* was outlined with the lowest number of nymphs and three-fold more numerous imagines. Again, female *B. caprae* exceeded the number of males at the highest extent (M: F - 1:7.2).

Out of the examined 4599 goats from 34 farms, 2608 goats from 29 farms were infested with one of more parasitic insect species.

The results about the type of infestation were also interesting. Data from Table-3 showed that among the 2608 infected goats, the share of hosts infested with more than a single species was the highest (75.31%). Multiple infestations were present in most of the surveyed farms (18 out of 29 affected, Table-3). In all 1964 goats with multiple infestations, the number of animals invaded simultaneously with 4 ectoparasitic insect species was the highest (39.26%, Table-3).

**Table-3:** Relative proportions of goats with monoinfestation and mixed infestation with ectoparasitic insects.

Objects studied	Total infested	Mono-infestation	Mixed infestation			

			Total	2 species	3 species	4 species
Goats	2608	644 (24.69%)	1964 (75.31%)	312 (11.96%)	628 (24.07%)	1024 (39.26%)
Farms	29	11	18	2	5	11

During the survey, the regional distribution of intensity and EI with detected ectoparasites on goats were also investigated. Table-4 shows that the mean II with bloodsucking *Linognathus* species was from 0 to 2930 insects. No infestation was found in 6 regions. The highest mean intensity of infection was recorded in Rousse (2930 insects), Sliven (2460 insects), and Blagoevgrad (1870 insects (Table-4). In the other 7 regions, the mean II varied from 360 (Pazardzhik) to 1640 insects (Stara Zagora). The average II for the country was 907 insects with EI of 31.7%.

**Table-4:** Regional distribution of the mean intensity and mean extensity of invasion with bloodsucking *Linognathus* species in domestic goats.

No.	Region	Number of studied goats	Number of infested goats	Mean intensity of invasion (number)	Mean extensity of invasion (95%CL)
1	Stara Zagora	272	89	1640	31.8 (26 ÷ 37)
2	Sofia	16	0	0	0 (0 ÷ 20)
3	Plovdiv	300	0	0	0 (0 ÷ 1)
4	Haskovo	1160	105	540	9.0 (7 ÷ 10)
5	Sliven	300	91	2460	30.3 (25 ÷ 35)
6	Yambol	300	218	1500	72.6 (67 ÷ 77)
7	Burgas	479	272	1270	56.7 (52 ÷ 61)
8	Pleven	54	0	0	0 (0 ÷ 6)
9	Blagoevgrad	680	363	1870	53.3 (49 ÷ 57)
10	Varna	16	8	590	50.0 (26 ÷ 73)
11	Veliko Tarnovo	264	213	1360	80.6 (75 ÷ 85)
12	Lovech	134	0	0	0 (0 ÷ 0)
13	Rousse	56	45	2930	80.3 (70 ÷ 90)
14	Targovishte	350	0	0	0 (0 ÷ 1)
15	Pazardzhik	116	57	360	35.4 (28 ÷ 42)
16	Razgrad	102	0	0	0 (0 ÷ 0)
Average for the country		4599	1461	907	31.7 (30 ÷ 33)

Infestation of goats with *B. caprae* was registered in 11 regions (Table-5). The highest II values were in Stara Zagora region (2680 insects), and the lowest in Rousse region (530 insects). The average II for the country was 694 insects, and EI – 28.9 %.

**Table-5:** Regional distribution of the mean intensity and mean extensity of invasion with *B. caprae* in domestic goats.

No.	Region	Number of studied goats	Number of infested goats	Mean intensity of invasion (number)	Mean extensity of invasion (95%CL)
1	Stara Zagora	272	122	2680	43.7 (37 ÷ 49)
2	Sofia	16	0	0	0 (0 ÷ 20)
3	Plovdiv	300	57	730	19.0 (14 ÷ 23)
4	Haskovo	1160	75	490	6.4 (5 ÷ 7)
5	Sliven	300	142	480	47.3 (41 ÷ 53)
6	Yambol	300	75	500	25.0 (20 ÷ 29)
7	Burgas	479	319	1950	66.5 (62 ÷ 70)
8	Pleven	54	0	0	0 (0 ÷ 6)
9	Blagoevgrad	680	209	1320	30.7 (27 ÷ 34)
10	Varna	16	15	990	93.7 (81 ÷ 100)
11	Veliko Tarnovo	264	249	840	94.3 (91 ÷ 97)
12	Lovech	134	0	0	0 (0 ÷ 0)
13	Rousse	56	15	530	26.7 (15 ÷ 38)
14	Targovishte	350	0	0	0 (0 ÷ 1)
15	Pazardzhik	116	54	600	33.5 (26 ÷ 40)
16	Razgrad	102	0	0	0 (0 ÷ 0)
Average for the country		4599	1332	694	28.9 (27 ÷ 30)

The comparison of data from Tables-4 and 5 demonstrated that the infestation with bloodsucking lice from genus *Linognathus* was more pronounced compared to that with biting lice from the genus *Bovicola*. This was confirmed both by the number of infested goats, and by II and EI values which were greater for *Linognathus* lice – 907 insects and EI 31.7% versus 694 insects and EI 28.9% for *Bovicola* biting lice.

Data for the infestation with *P. irritans*, the only flea species detected on goats are presented in Table-6. This ectoparasite was present in 11 out of the 16 surveyed regions in Bulgaria. The DI ranged from 1 insect (Yambol region) to 5 insects (Stara Zagora, Haskovo and Blagoevgrad regions). The EI of *P. irritans* (32.3%) is highly indicative for the spread of the parasite; it may be compared to that of the bloodsucking lice from the genus *Linognathus* (31.7%) (Tables-4 and 6).

**Table-6:** Regional distribution of the degree of infestation and mean extensity of invasion with *P. irritans* in domestic goats.

No.	Region	Number of studied goats	Number of infested goats	Degree of infestation	Mean extensity of invasion (95%CL)
1	Stara Zagora	272	10	5	3.5 (1 ÷ 5)
2	Sofia	16	0	0	0 (0 ÷ 20)
3	Plovdiv	300	57	4	19.0 (14 ÷ 23)
4	Haskovo	1160	310	5	26.7 (24 ÷ 29)
5	Sliven	300	62	2	20.6 (16 ÷ 25)
6	Yambol	300	18	1	6.0 (3 ÷ 8)
7	Burgas	479	235	4	49.0 (44 ÷ 53)
8	Pleven	54	25	2	46.2 (33 ÷ 59)
9	Blagoevgrad	680	611	5	89.8 (87 ÷ 92)
10	Varna	16	0	0	0 (0 ÷ 0)
11	Veliko Tarnovo	264	0	0	0 (0 ÷ 0)
12	Lovech	134	73	3	54.4 (46 ÷ 62)
13	Rousse	56	0	0	0 (0 ÷ 0)
14	Targovishte	350	85	2	24.2 (19 ÷ 28)
15	Pazardzhik	116	0	0	0 (0 ÷ 0)
16	Razgrad	102	0	0	0 (0 ÷ 0)
Average of the country		4599	1486	2	32.3 (31 ÷ 33)

## Discussion

The aim of the present study was to identify the species of ectoparasitic insects on domestic goats and the population structure, intensity and EI with found species. Its results would facilitate the diagnosis, prognosis of future economic losses and implementation of timely measures for control of these entomoses.

The lice species *L. stenopsis*, *L. africanus*, and *B. caprae* are globally spread [2]. Our study confirmed that they were also widely spread in Bulgaria with relatively similar rates in different regions. The lack of these species in some regions may be attributed to the relatively small number of examined settlements and farms. Furthermore, all farms that were free of lice have declared a recent anti-parasitic treatment with preparations effective against these species. Goats are common flea hosts. Some researchers reported infestations with *P. irritans* in countries with moderate climate [6]. Detailed and large-scale studies on flea species infesting goats in Bulgaria have not been conducted so far. This study found the species *P. irritans* as the only representative of all flea species. Its spread, however, was highly variable from a regional point of view. This may be explained by the fact that the temporary parasitism by fleas makes them strongly dependent on abiotic environmental conditions. *Hippobosca equina* is not a highly host-specific parasite encountered in Europe and Bulgaria [27], so its occurrence in goats from our country was expected.

Our results on population structure were confirmed by Kumar *et al*. [28] and Santos *et al*. [11], reporting a higher number of female *B. caprae* imagines than that of males. A similar sex ratio was observed for representatives of the family Linognathidae. It was 1:3.5 for *L. stenopsis* with adult: numph (A: N) ratio of 1:0.45. In sheep from India, Rashmi and Saxena [29] found a male-to-female (M: F) ratio of 1:2 for *L. ovillus* e 1:2, with A: N ratio of 1:2.3. For *L. pedalis*, M: F was 1:1.5, and A:N − 1:1.2. These authors affirmed that in this species, female insects were more abundant than males, in agreement with our results. Our survey showed a lower number of nymphs versus imagines, contrary to other published studies. This discrepancy was probably due to the different study seasons. Data on sex-related structure of *P. irritans* population showed predominance of male insects over females (1:0.8), whereas in goats, Dipeolul and Ayoade [30] reported that infestation with *C. felis strongylus* was mainly with female insects (52:25). The possible reason for this finding is the temporary parasitism of these insects. Out of the six ectoparasitic species detected on goats in the country, only fleas exhibited a slight predominance of the number of males over that of females, probably because a large part of adult insects are in the environment and not on the host. In all other insects, the females were more numerous.

Data from Table-3 demonstrated that multiple infestation with biting and sucking lice was very common (in 16 of surveyed farms). *Linognathus stenopsis* monoinfestation but no *B. caprae* was detected only at one of the 11 farms. This finding supported an earlier observation of Nedelchev [4], who reported a mixed *L. stenopsis* and *B. caprae* infestation in 22 (71.5%) of studied affected herds. This may be explained by the lack of competition among lice-infesting goats, as members of various ecological niches.

The mean II of goats with ectoparasites is a highly variable parameter as seen from both out (907 *Linognathus* spp.; 694 *B. caprae* and 3−6 *P. irritans*), and from other studies [4, 15, 16]. The factors influencing the intensity of infection are numerous, yet may be generally reduced to age of infestation and host age and health status. Thus, Fivaz *et al*. [31] found out that 2-day-old goat kids were already infested with *D. limbata*, and 5 months later, the mean II increased from 13 to 3,994. Kusiluka *et al*. [32] reported that goat kids were massively infested with *C. felis* unlike adult goats. Climatic conditions and season are other factors that influence infestation intensity. Nedelchev [4] demonstrated that *B. caprae* and *L. stenopsis* in goats had the highest II in January-February and the lowest – during the summer. Suboptimal rearing conditions during the winter (low ambient temperature, high humidity, poorer hygiene, deficient feeding, and parturition during that season) compromise the immunity of animals and presume a more intensive infestation with parasites, in support of the increased intensity of infection during the winter.

The average country EI with lice may be assumed as a representative, because the EI with bloodsucking lice for Stara Zagora region (where the number of examined herds was the greatest) was very close (31.8% and 31.7%, respectively). The average EI with fleas was the highest compared to values for the other detected insect species. This may be attributed to the great motility and adaptability of fleas. Their spread is ubiquitous and the absence in 6 of studied regions may be probably due to the small number of examined animals, regular anti-parasitic treatment and manure cleansing, as well as rearing of herds in highland and foothill regions. That is why the greatest EI with fleas was registered in the southernmost from surveyed regions in Bulgaria – Blagoevgrad. Similar values for EI with fleas on goats were reported by Shuvo *et al*. [5] and Aboulqassim *et al*. [33].

## Conclusion

The performed survey on species composition and spread of ectoparasitic insects on domestic goats in Bulgaria detected six insect species infesting domestic goats: *Linognathus stenopsis* Burmeister, 1838, *L. africanus* Kellog and Paine, 1911, *B. caprae* Gurlt, 1843, *P. irritans* Linnaeus, 1758, *H. equina* Linnaeus, 1758 and *L. cervi* Linnaeus, 1758. This study found the species *P. irritans* as the only representative of all flea species. The species *L. stenopsis*, *L. africanus*, *B. caprae*, and *P. irritans* were the most prevalent amount domestic goat herds in the country. They were detected in more than 40% of examined farms located in 68.75% of surveyed regions in Bulgaria.

## Author’s Contributions

NSN: Conceived the work, conducted the study, researched the literature, and drafted and revised the manuscript. The author has read, reviewed, and approved the final manuscript.
